# The impact of Sjӧgren’s syndrome on the quality of sexual life of female patients in the UK: a controlled analysis

**DOI:** 10.1007/s00296-021-04830-6

**Published:** 2021-03-10

**Authors:** Minan Al-Ezzi, Anwar R. Tappuni, Khalid Saeed Khan

**Affiliations:** 1 Centre of Teaching and Innovation, Institute of Dentistry, Barts and The London School of Medicine and Dentistry, Queen Mary University of London, London, UK; 2 College of Medicine and Dentistry, Ulster University, Birmingham, UK; 3 Barts and The London School of Medicine and Dentistry, Queen Mary University of London, London, UK; 4 Department of Preventive Medicine and Public Health, Faculty of Medicine, University of Granada, Granada, Spain

**Keywords:** Sexual function, Primary Sjӧgren’s syndrome, Dyspareunia, Vaginal dryness, Quality of life, Fatigue

## Abstract

Mucosal dryness and dyspareunia are symptoms that may significantly affect women with primary Sjӧgren syndrome (pSS). We investigated whether vaginal dryness is correlated with sexual function, and the impact may have on the quality of life (QoL) and mental health well-being in pSS patients. Ethically approved comparative cross-sectional study was designed to assess sexual function using the Female Sexual Function Index (FSFI) in 65 pSS female patients vs 62 sex-matched controls. The effect of vaginal dryness and fatigue on sexual function was investigated. Vaginal dryness was correlated with oral dryness estimated by salivary flow rate and the Clinical Oral Dryness Score to investigate whether genital dryness is indicative of general mucosal dryness in pSS. Validated questionnaires were used to investigate the effect of sexual function on QoL and mental health well-being. The number of sexually active pSS participants was significantly less than in the control group (28/65 vs 42/62, *p* < 0.05). The sexual function was significantly impaired in the pSS group (mean FSFI = 19 vs 28.3, *p* < 0.05). There was no significant association between self-reported vaginal dryness and oral dryness or sexual function. The open-ended questions showed that the most troublesome symptom reported by pSS patients was oral dryness (43%, *n* = 28/65) followed by fatigue (31%, *n* = 20/65). Sexual dysfunction had a negative impact on QoL and the mental health well-being of pSS patients in all aspects, especially on the quality of social life (*β* = 0.7, *p* = 0.02). Addressing sexual dysfunction can potentially improve the QoL of pSS patients significantly, especially their social well-being.

## Introduction

Vaginal dryness and dyspareunia are purported to be symptoms in women with primary Sjӧgren’s syndrome (pSS), but there is limited evidence on whether these symptoms have significant effect on the sexual function and/or on the quality of life (QoL) of pSS patients [1]. More than one element can influence the sexual function in Sjӧgren’s syndrome (SS) patients, including joints pain, and age and sex hormones as reported in rheumatic patients [2, 3]. Although vaginal dryness is a recognised symptom in pSS, the literature has suggested variable impact of this on the sexual function and mental health well-being [4–6]. Recent studies have emphasised that vaginal dryness can play significant role in deteriorating the sexual life of pSS patients [7, 8]. However, other studies showed no correlation between sexual activity and deteriorated vaginal lubrication, oral, or lacrimal dryness in pSS patients [9, 10]. There is also evidence that psychological factors have stronger influence on sexuality than physical determinants [2, 10]. Dyspareunia in SS patients was found to be associated with factors other than dryness, such as trauma or inflammation [11]. Other studies reported that any chronic disease associated with fatigue syndrome can influence sexual relationships [12–14]. In conclusion, there is no consensus in the literature concerning sexual dysfunction in women with pSS. In the current study, we investigated whether the sexual function in a well-defined group of female pSS patients is compromised compared with healthy controls. The frequency of self-reported vaginal dryness in pSS patients and its correlation with dryness of the mouth was examined. The effect of reduced sexual function on QoL and mental health well-being was studied.

## Methodology

A comparative study was ethically approved by the Research Ethical Committee (Reference number 15/LO/2064, 10/02/2016). The study was structured according to STROBE statement checklist for combined studies (case–control and cross-sectional studies) [15].

The sexual activity was assessed with the Female Sexual Function Index (FSFI), which is a validated 19-item questionnaire distributed into six domains: desire, arousal, lubrication, orgasm, satisfaction, and pain [16]. The total score of the questionnaire is 36, where a higher score denotes better sexual function. A cut-off value of ≤ 26.55 was adopted from a previous study to identify subjects who are at risk of sexual dysfunction [17]. Vaginal dryness was subjectively assessed by asking patients whether or not they suffer from this symptom. Visual analogue scale (VAS) was used for fatigue self-rating with an arbitrarily specified cut-off value of 50, any higher ratings indicate poor assessment over a 100 graded scale. The oral dryness severity was assessed by stimulated and unstimulated salivary flow rate (SFR), and by the Clinical Oral Dryness Score (CODS) [18–20].

The general QoL was assessed by (WHOQoL-BRÉF), and the mental health well-being was assessed by Hospital Anxiety and Depression Scale (HADS) [21, 22]. Uncoded items were checked, and if applicable, participants were requested to complete the questionnaire forms. The questionnaires were considered invalid if more than 20% of the data were missing.

### Study group

The study was conducted in the Multidisciplinary Sjӧgren’s Syndrome Clinic, Institute of Dentistry, Barts Health Trust in East London. Patients were recruited from the above clinic or identified from the SS research clinical database of 337 patients. The database was screened for eligible patients who were defined as women > 18 years old and diagnosed with pSS according to the American–European Consensus Group (AECG) criteria [23]. If patients’ diagnosis was unclear on the database, the blood test results and the medical records were reviewed to clarify the diagnosis. Postal invitations modified from Dillman methodology were sent to 122 eligible patients identified as above [24]. The project was also advertised on the British Sjögren’s syndrome Association website and interested pSS members were invited to take part.

Sex-matched healthy volunteers were recruited for comparison, in a ratio of 1:1, by advertising the project in the Institute of Dentistry via posters, recruitment leaflets, and information sheets. Subjects were excluded if they have had medications that can cause vaginal dryness, head and neck or lower abdomen radiation treatment, chemotherapy, chronic salivary gland disease or swelling, secondary SS, asthma, allergic sinusitis, cold/blocked nose, uncontrolled diabetes, pregnancy, breast feeding, candidiasis, lichen planus, or significant dental problems.

### Statistical analysis

Data was analysed using the latest version of Statistical Package for Social Sciences, IBM Corporation, SPSS Inc., Chicago, IL, USA version-23 statistical software. A pilot study was conducted to help estimate the sample size calculation that was based on the outcome of a larger study [25]. The power was set at 90% and the level of significance at 5%. A total of at least 75 subjects (cases and healthy volunteers) would be required to detect a level of difference. To accommodate for dropout, the sample was inflated by 30% of participants. Continuous variables were expressed as mean difference followed by 95% confidence interval (CI). Independent *t* test, Chi-square, and multiple regression analysis tests were used. Residual plots were used to assess the quality of regression. Frequency analysis was used to determine the rate of the self-reported symptoms affecting patients’ QoL. Binary logistic regression was used to predict the self-reported vaginal dryness (dependant variable) from oral dryness measures (independent variable) and to control confounders such as age, alcohol, and disease duration. The analysis was only carried out in the subject group. All *p* values were reported for transparency regardless of significance.

## Results

### Sexual function

**Fig. 1:**
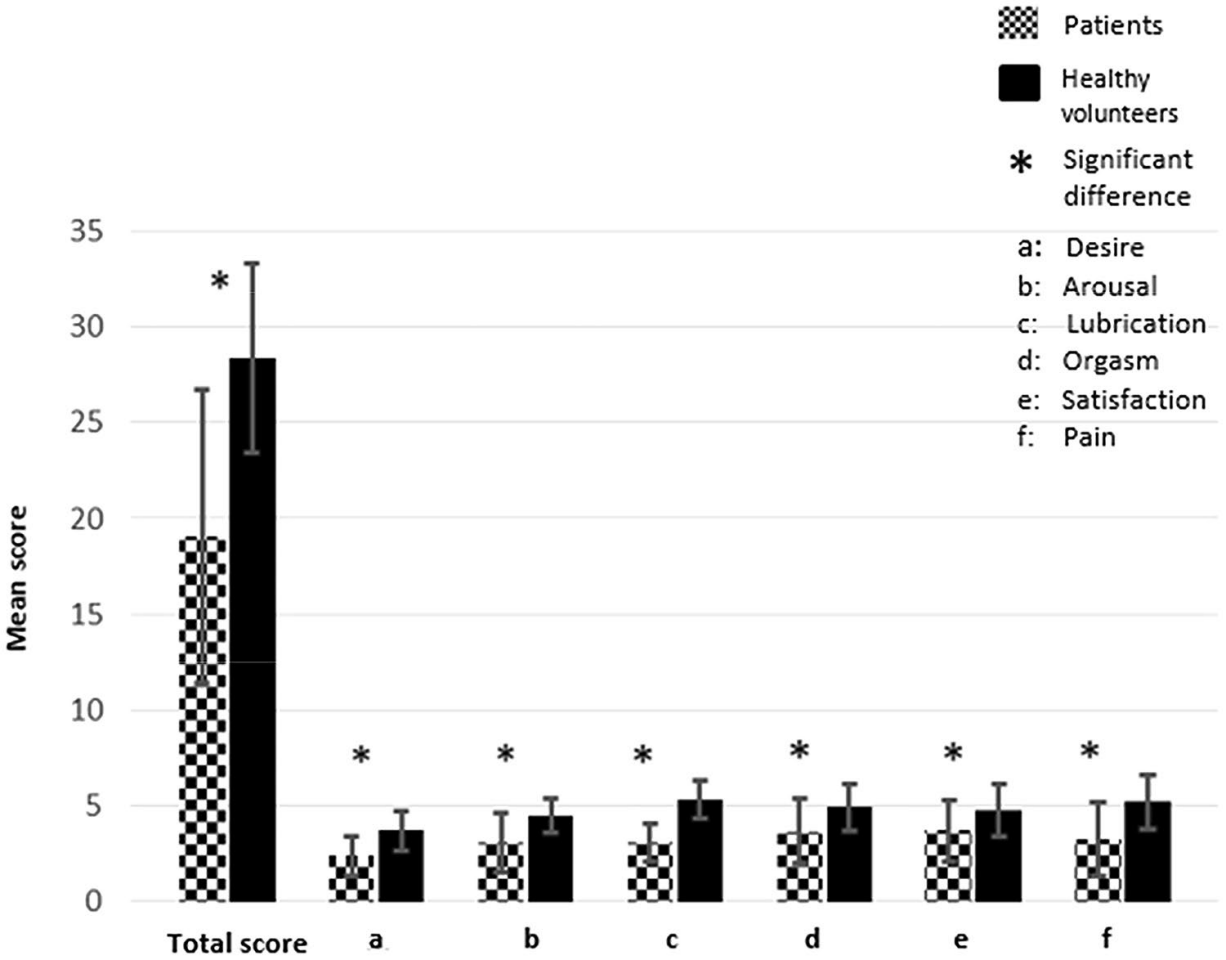
Comparison of the mean difference of FSFI global and its domains between pSS patients and healthy controls. Bars indicates SD

Sixty-five eligible pSS patients and 62 sex-matched healthy volunteers gave consent to participate in the study. The most common ethnic origin was “white” in the patients (69%) and in the control (45%) groups. Out of whom, 28 patients and 42 controls were sexually active and therefore were included in the statistical analysis. In the FSFI, the score of zero of an individual domain indicates that the subject reported having no sexual activity during the past 4 weeks. Any values above zero indicate sexual functionality. Participants with sexual dysfunction in the pSS and controls groups were 82.1% (*n* = 23/28) and 33.3% (*n* = 14/42), respectively, as measured by FSFI (mean difference = 9.2, *p* < 0.05). The total score of the sexual function was decreased significantly in the sexually active pSS patients (19.1, ± 7.7) compared with the sexually active controls (28.4, ± 4.9). The mean difference of the total score was 9.3, 95% CI 6–12.6 (Fig. 1). Desire scored the lowest among all FSFI domains in the pSS group compared with controls (mean difference = 1.3, *p* < 0.05) (Table 1; Fig. 1).

**Table 1:** Comparison of the FSFI domains in each group

FSFI domains	pSS patients Sexually active Mean age (%95%) 59 (55.8–62.1)	Healthy volunteers Sexually active Mean age (%95%) 43 (39.2–46.8)	Mean difference (95% CI)	*p* value
	Mean Minimal scoring rate, n of minimal scoring	Mean Minimal scoring rate, n of minimal scoring		
Desire (score range 1.2–6)	2.38 32%, *n* = 9/28	3.7 2.4%, *n* = 1/42	1.3 (0.8–1.8)	< 0.05
Arousal (score range 0–6)	3.1 0%, *n* = 28/28	4.4 0%, *n* = 42/42	1.3 (0.7–2)	< 0.05
Lubrication (score range 0–6)	3.1 3.6%, *n* = 1/28	5.2 0%, *n* = 42/42	2.1 (1.5–2.7)	< 0.05
Orgasm (score range 0–6)	3.6 0%, *n* = 28/28	4.9 0%, *n* = 42/42	1.2 (0.5–2)	< 0.05
Satisfaction (score range 0.8–6)	3.7 0%, *n* = 28/28	4.7 0%, *n* = 42/42	1 (0.3–1.7)	< 0.05
Pain (score range 0–6)	3.2 6.7%, *n* = 2/28	5.2 2.4%, *n* = 1/42	1.9 (1.1–2.7)	< 0.05
Total score	19 82.1%, *n* = 23/28	28.3 33.3%, *n* = 14/42	9.23 (6–12.6)	< 0.05

The open-ended questions showed that the symptoms of burden that were frequently reported by pSS patients were oral dryness (43%, *n* = 28/65) and fatigue (31%, *n* = 20/65). Whilst joint pain (8%, *n* = 5/65) and vaginal dryness (2%, *n* = 1/65) were reported as less troublesome symptoms.

### Self-reported vaginal dryness in pSS group

The self-reported vaginal dryness was present in 87% (*n* = 54/62) of pSS patients who answered the question of whether they suffer from vaginal dryness. Out of the 28 sexually active pSS patients, 26 (92.8%) reported vaginal dryness, one patient declined to answer, and one patient reported no vaginal dryness. The sexually inactive patients were 37, out of whom 75.6% (*n* = 28) reported dry vagina.

### Self-reported vaginal dryness impact on the sexual function in pSS patients

In the sexually active patients (*n* = 26), vaginal dryness did not correlate significantly with any of the FSFI domains. Fatigue as measured by VAS had correlated with the global score of FSFI, but did not reach significance level (*β* = − 0.3, 95% CI − 20–5) (Table 2).

**Table 2:** Coefficients’ table of the correlation between self-reported vaginal dryness and the sexual function measured by FSFI domains in the sexually active pSS patients

Vaginal dryness	Standardized coefficients (*β*)	*p* value	95% CI
Desire	− 0.1	0.5	− 3–1.6
Arousal	0.04	0.8	− 3–4
Lubrication	0.05	0.8	− 3–3.6
Orgasm	0.2	0.5	− 2.4–5.2
Satisfaction	0.2	0.4	− 2–4.6
Pain	− 0.2	0.3	− 6.2–2
FSFI global	0.006	0.9	− 16–17

### Oral dryness and self-reported vaginal dryness

Oral dryness was found in 78.4% (*n* = 51/65) in pSS patients using USFR, 64.6% (*n* = 42/65) using SSFR, and 84.6% (*n* = 55/64) using CODS. No significant correlation was found between oral and vaginal dryness (Table 3).

**Table 3:** Correlation between USFR, SSFR, or CODS with the self-reported vagina dryness in pSS group using logistic regression analysis

Oral dryness tests	Sexual^a^ function	*p* value	95% CI
USFR^b^	0.7	0.8	0–2
SSFR^c^	− 0.5	0.4	0.2–2
CODS^d^	0.8	0.7	0.7–1.6

^a^Measured by FSFI

^b^Unstimulated salivary flow rate

^c^Stimulated salivary flow rate

^d^Clinical oral dryness score

### Sexual dysfunction impact on quality of life and mental health well-being

Among the WHOQoL-BRÉF domains, sexual function had significant correlation with the social domain only in the sexually active patients (*β* = 0.6, 95% CI 0.3–3.3). There were no other significant correlations between sexual function and the other WHOQoL-BRÉF domains (physical, mental, and environmental). Additionally, anxiety and depression were not correlated with the impaired sexual function in the sexually active pSS patients (Table 4).

**Table 4:** Sexual dysfunction impact on the quality of life and mental health well-being in pSS patients

QoL	Standardized coefficients (*β*)	*p* value	95% CI
Physical domain (WHOQoL-BRÉF)	0.3	0.4	− 0.9–2.2
Psychological domain (WHOQoL-BRÉF)	0.4	0.2	− 0.4–2.2
Social domain (WHOQoL-BRÉF)	0.7	0.02	0.4–3.3
Environmental domain (WHOQoL-BRÉF)	0.3	0.4	− 0.7–1.9
Anxiety (HADS)	− 0.6	0.08	− 0.6–0.04
Depression (HADS)	− 0.6	0.06	− 0.6–0.01

## Discussion

This study, designed to assess the sexual function in pSS patients, demonstrated that sexual dysfunction was as twice as prevalent in the pSS group as that in the control group. Vaginal dryness was not the key indicative factor for sexual deterioration as was suggested in some studies in the literature [4, 5]. Our results did not support that dryness of different mucosal sites does necessarily manifest simultaneously in pSS patients. Sexual dysfunction had a negative impact on the social QoL of pSS patients but not on the physical or the psychological domains. Interestingly, patients were mostly bothered by oral dryness and fatigue rather than by vaginal dryness or joint problems.

The study was structured according to STROBE checklist for combined studies to ensure clear presentation of the design, conduct, and the results of this observational study. The power calculation (90%) and the sample size (patients *n* = 65, controls *n* = 62) were large enough to come up with a conclusion. However, the power calculation was based on the mean difference of the primary outcomes only; hence, unpowered regression analysis for the patients’ group was obtained. All *p* values were reported for transparency regardless of significance. However, the correlations cannot be overlooked even without reaching the significance level.

Patients were diagnosed by a specialist member of a multidisciplinary team utilising the AECG criteria. This ensured identifying a well-defined group of patients for the study. However, it is recognised that the study group may represent the more severe spectrum of the disease, as the patients were recruited from a specialised clinic in a tertiary referral centre. To avoid performance bias, data were collected by one researcher. One of the shortcomings of this study is the discrepancy between the control group mean age (43 years) and the pSS group (59 years), which may have influenced the sexual activity data. Therefore, to validate our results, we analysed the data of a subgroup of our study subjects of the same age range of that of the control group and found that the results remained the same as the overall analysis (results are not presented). A limitation of the FSFI questionnaire reported by several of our participants, who stated that the 4 week period limited by the questionnaire in assessing women’s sexual activity, is not representative. Some participants commented that their sexual life has just changed with recently changed circumstances rather than due to health reasons or declined desire. It should be acknowledged that the FSFI is not a diagnostic tool but a brief questionnaire for the assessment of women’s sexual function. Therefore, a more comprehensive tool to assess sexual function over longer time period may be more useful in sexual activity studies.

We found that half of our pSS patients were sexually inactive which was higher than the previously reported figures [4]. Furthermore, using a cut-off score of 26.5 in FSFI. A total of 23/28 patients (82.1%) and 14/42 healthy volunteers (33.3%) were considered to have impaired sexual function, which is comparable to a previous report where 83.3% patients and 37.5% controls reported sexual dysfunction [5]. The obvious impairment of the sexual function in the pSS group compared with controls is an indication in itself of the impact of the syndrome on sexuality. We found that all FSFI domains were affected in women with pSS and showed sexual impairment compared with controls, including the ability to lubricate and pain sensation, in line with findings reported in the literature [4, 5].

Vaginal dryness was subjectively assessed in this study as it was self-reported. This approach was selected, because it was more acceptable to patients compared with the more accurate but more invasive methods of having a vaginal examination. In the subgroup analysis of the FSFI, stratified according to the sexually active patients using multiple regression models, we found that the self-reported vaginal dryness did not play a key role in compromising the global FSFI score. These results agreed with others who concluded that the sexual function and the frequency of intercourse in pSS were not correlated with the dry vagina [2, 10, 11]. Anecdotally, clinicians report that vaginal dryness is a frequently reported symptom of pSS. In line with this, a large proportion of our sexually active (92.8%, *n* = 26/28) and sexually inactive patients (75.6%, *n* = 28/37) reported vaginal dryness. Although vaginal dryness was common in our study group, it did not affect sexual activity. In a study of a large cohort of systemic lupus erythematosus (*n* = 302), 30% of the subjects had accompanied SS with vaginal dryness reported sexual dysfunction [26]. However, their FSFI scores were not significantly different from those without SS which indicates that vaginal dryness was not a major factor of the sexual problems. Interestingly, our open-ended questions revealed that vaginal dryness was not the most bothersome symptom that affected patients’ QoL. In fact, only one patient out of 65 (1.5%, *n* = 1/65) had reported dry vagina as the worst symptom of the syndrome. It is possible that due to the sensitivity of the subject, vaginal dryness is under reported. In the current study, some patients reported painful intercourse despite using artificial lubricants and that majority of patients who declared having sexual dysfunction, did not cite vaginal dryness as a reason, but 30% attributed it to fatigue (*n* = 20/65). This may suggest that physical pain represented by fatigue and/or joint stiffness rather than vaginal dryness is the effector in sexual function.

Our findings were compatible with other studies where no association was found between vaginal dryness and dyspareunia in 21 pSS patients diagnosed by the European criteria [2]. This is not surprising as dyspareunia was also reported in premenopausal pSS patients with healthy vaginal mucosa in a study of 51 SS patients [11]. Therefore, our results support others in that the sexual activity of pSS patients is driven by the severity of fatigue rather than vaginal dryness [4, 12–14].

The open-ended questions highlighted aspects that were not covered by the routine clinical tests and the questionnaires used in the study. In fact, there appears to be a suggestion of more than one element that can influence sexual function in rheumatic patients including joint pain, and age and sex hormones. Therefore, there is merit in evaluating patients’ sexual problems through semi-structured interviews with predetermined topics, to explore in greater depth the issues raised which will help develop referral pathway for affected patients.

Oral dryness was statistically significantly higher in the pSS group than in the healthy volunteers. However, in the logistic regression analysis, a correlation was established between oral dryness and the self-reported vaginal dryness, but this did not reach statistical significance. Therefore, powered regression analysis is recommended to verify these findings. To our knowledge, this is the first time that this relationship has been studied.

We found that sexual dysfunction was a common debilitating factor that interfered with the general QoL of patients in all four domains despite of the insignificant contribution in the physical, psychological, and environmental domains, in agreement with our previous findings [1]. The pSS patients were more anxious and four times more depressed than the healthy volunteers. Our results were in line with the other studies where the mental health well-being was affected significantly in pSS patients [6, 27–30]. We found that anxiety symptoms were higher in pSS patients but not significantly different from that of the controls. Whereas depression was significantly higher in our pSS group compared with that in controls. These findings supported others who reported that depression symptoms were pronounced more than anxiety in pSS patients when assessed by HADS [4, 27, 30]. Sexual dysfunction compromised QoL and mental health status in the pSS group; however, a study with powered regression analysis is needed to confirm these findings.

In summary, the evidence from our study and previous studies demonstrate that sexual dysfunction is a multi-factorial problem in pSS patients, which may be caused by fatigue and joint pain rather than vaginal dryness only. In this study, we present valuable evidence to indicate that sexual impairment is a symptom in pSS, which significantly compromises patients’ social QoL. The self-reporting of dissatisfaction with personal relationships, social support, and sexual activity were in line with this conclusion. This novel finding highlights the importance of sexual satisfaction for the perception of living a full active social life. However, the clinical management of pSS in most UK clinics at present does not include routine formal assessment of patients’ sexual activity. The introduction of an assessment tool including relevant questionnaire to recognise sexual impairment and to establish an appropriate referral pathway for affected individuals would improve the clinical service provision for these patients and may significantly improve their QoL.

## References

[1] AL-Ezzi MY, Pathak N, Tappuni AR, Khan KS (2017) Primary Sjögren’s syndrome impact on smell, taste, sexuality and quality of life in female patients: a systematic review and meta-analysis. Mod Rheumatol 27:623–629. https://www.tandfonline.com/doi/abs/10.1080/14397595.2016.1249538 10.1080/14397595.2016.1249538 27760487

[2] Valttysdottir ST, Wide L, Hallgren R (2003) Mental well-being and quality of sexual life in women with primary Sjögren’s syndrome are related to circulating dehydroepiandrosterone sulphate. Ann Rheum Dis 62:875–879. https://ard.bmj.com/content/62/9/875.short 10.1136/ard.62.9.875 PMC1754646 12922962

[3] Tristano AG (2009) The impact of rheumatic diseases on sexual function. Rheumatol Int 29(8):853–860. https://link.springer.com/article/10.1007/s00296-009-0850-6 10.1007/s00296-009-0850-6 19152092

[4] Van Nimwegen JF, Arends S, Van Zuiden GS, Vissink A, Kroese FG, Bootsma H (2015) The impact of primary Sjögren’s syndrome on female sexual function. Rheumatology 54:1286–1293. https://academic.oup.com/rheumatology/article/54/7/1286/1851813?login=true 10.1093/rheumatology/keu522 25652072

[5] Priori R, Minniti A, Derme M, Antonazzo B, Brancatisano F, Ghirini S et al (2015) Quality of sexual life in women with primary Sjögren syndrome. J Rheumatol 42:1427–1431. https://www.jrheum.org/content/42/8/1427.short 10.3899/jrheum.141475 26136488

[6] Ugurlu GK, Erten S, Ugurlu M, Caykoylu A, Altuoglu A (2014) Sexual dysfunction in female patients with primary Sjögren’s syndrome and effects of depression: cross-sectional study. Sex Disabil 32:197–204. https://link.springer.com/article/10.1007%2Fs11195-014-9352-x

[7] Yildis Ç, Karakus S, Bozoklu Akkar Ö, Şahin A, Bozkurt B, Yanik A (2017) Primary Sjögren’s syndrome adversely affects the female sexual function assessed by the female sexual function index: a case-control study. Arch Rheumatol 32(2):123–128. https://www.ncbi.nlm.nih.gov/pmc/articles/PMC6190991/10.5606/ArchRheumatol.2017.6066 PMC6190991 30375566

[8] Isik H, Isik M, Aynioglu O et al (2017) Are the women with Sjögren’s syndrome satisfied with their sexual activity?. Rev Bras Reumatol Engl Ed 57(3):210–216. http://acikarsiv.beun.edu.tr/xmlui/handle/20.500.12628/4345 10.1016/j.rbre.2017.01.002 28535892

[9] Mulherin DM, Sheeran TP, Kumararatne DS, Speculand B, Luesley D, Situnayake RD (1997) Sjögren’s syndrome in women presenting with chronic dyspareunia. Br J Obstet Gynaecol 104:1019–1023. https://obgyn.onlinelibrary.wiley.com/doi/abs/10.1111/j.1471-0528.1997.tb12060.x 10.1111/j.1471-0528.1997.tb12060.x 9307528

[10] Marchesoni D, Mozzanega B, De Sandre P, Romagnolo C, Gambari PF, Maggino T (1995) Gynaecological aspects of primary Sjögren’s syndrome. Eur J Obstet Gynecol 63:49–53. https://www.sciencedirect.com/science/article/abs/pii/030121159502224U 10.1016/0301-2115(95)02224-u 8674565

[11] Skopuli FN, Papanikolaou S, Malamou-mitsi V, Papanikolaou N, Moutsoulos HM (1994) Obstetric and gynaecological profile in patients with primary Sjögren’s syndrome. Ann Rheum Dis 53:569–573. https://pubmed.ncbi.nlm.nih.gov/7979594/10.1136/ard.53.9.569 PMC1005407 7979594

[12] Goodwin SS (1997) The marital relationship and health in women with chronic fatigue and immune dysfunction syndrome: views of wives and husbands. Nurs Res 46:138–146. https://pubmed.ncbi.nlm.nih.gov/9176503/10.1097/00006199-199705000-00004 9176503

[13] Frikha F, Maazoun F, Ben Salah R, Snoussi M, Masmoudi J, Nabil Mhiri M et al (2011) Sexual function in married women with rheumatoid arthritis. Presse Med 40:e521–e527. https://pubmed.ncbi.nlm.nih.gov/21763097/10.1016/j.lpm.2011.04.015 21763097

[14] Blazquez A., Ruiz E, Alistel L, Garcia-Quintan A, Alegre J (2015) The effect of fatigue and fibromyalgia on sexual dysfunction in women with chronic fatigue syndrome. J Sex Marital Ther 41:1–10. https://www.tandfonline.com/doi/abs/10.1080/0092623X.2013.864370 10.1080/0092623X.2013.864370 24274008

[15] Fernandez E (2005) Observational studies in epidemiology (STROBE). Med Clin 125(Supplement 1):43–48. https://www.sciencedirect.com/science/article/abs/pii/S0025775305722090 10.1016/s0025-7753(05)72209-0 16464427

[16] Rosen RC, Brown, C., Heiman, J et al (2000) The Female Sexual Function Index (FSFI): A multidimensional self-report instrument for the assessment of female sexual function. J Sex Marital Ther 26:191–208. https://www.tandfonline.com/doi/abs/10.1080/009262300278597 10.1080/009262300278597 10782451

[17] Wiegel M, Meston C, Rosen R (2005) The female sexual function index (FSFI): cross-validation and development of clinical cut-off scores. J Sex Marital Ther 31:1–20. https://www.tandfonline.com/doi/abs/10.1080/00926230590475206 10.1080/00926230590475206 15841702

[18] Navazesh M, Cheristensen CM (1982) A comparison of whole mouth resting and stimulated salivary measurement procedures. J Dent Res 61:1158–1162. https://journals.sagepub.com/doi/abs/10.1177/00220345820610100901 10.1177/00220345820610100901 6956596

[19] Navazesh M (1993) Methods for collecting saliva. Ann N Y Acad Sci 694:72–77. https://nyaspubs.onlinelibrary.wiley.com/doi/abs/10.1111/j.1749-6632.1993.tb18343.x 10.1111/j.1749-6632.1993.tb18343.x 8215087

[20] Osailan SM, Pramanik R, Shirlaw P, Proctor GB, Challacombe SJ (2012) Clinical assessment of oral dryness: development of a scoring system related to salivary flow and mucosal wetness. Oral Surg Oral Med Oral Pathol Oral Radiol 114:597–603. https://www.sciencedirect.com/science/article/abs/pii/S2212440312003914 10.1016/j.oooo.2012.05.009 22959491

[21] The WHOQOL Group (1998) Development of the World Health Organization WHOQOL-BREF quality of life assessment. Psychol Med 28:551–558.https://www.cambridge.org/core/journals/psychological-medicine/article/abs/development-of-the-world-health-organization-whoqolbref-quality-of-life-assessment/0F50596B33A1ABD59A6605C44A6A8F30# 10.1017/s0033291798006667 9626712

[22] Zigmond AS, Snaith RP (1983). The hospital anxiety and depression scale. Acta Psychiatr Scand.

[23] Vitali C, Bomardieri S, Jonsson R, Moutsopoulos HM, Alexander EL, Carsons SE (2002) Classification criteria for Sjögren’s syndrome: a revised version of the European criteria proposed by the American–European Consensus Group. Ann Rheum Dis 61:554–558. https://ard.bmj.com/content/61/6/554 10.1136/ard.61.6.554 PMC1754137 12006334

[24] Dillman DA (1983) Mail and self-administered surveys. In: Rossi PH, Wright JD, Anderson AB (eds) Handbook of survey research. Vol 10, pp 359–377

[25] Al-Ezzi, M, Khan K, Tappuni AR (2020) Is the taste acuity affected by oral dryness in primary Sjögren’s syndrome patients?. Oral Dis 26:688–695. https://pubmed.ncbi.nlm.nih.gov/31856365/10.1111/odi.13259 31856365

[26] Tseng JC, LU LY, HU JC, Wang LF, Yen LJ, WU HC (2011) The impact of systemic lupus erythematosus on women’s sexual functioning. J Sex Med 8:3389–3397. https://www.jsm.jsexmed.org/article/S1743-6095(15)33367-1/abstract 10.1111/j.1743-6109.2011.02464.x 21951616

[27] Stevenson HA, Jones ME, Rostron JL, Longman LP, Field EA (2004) UK patients with primary Sjögren’s syndrome are at increased risk from clinical depression. Gerodontology 21:141–145. https://pubmed.ncbi.nlm.nih.gov/15369016/10.1111/j.1741-2358.2004.00027.x 15369016

[28] Inal V, Kitapcioglu G, Karabulut G, Keser G, Kabasakal Y (2010) Evaluation of quality of life in relation to anxiety and depression in primary Sjögren’s syndrome. Mod Rheumatol 20:588–597. https://pubmed.ncbi.nlm.nih.gov/20585824/10.1007/s10165-010-0329-z 20585824

[29] Bongi SM, Del Rosso A, Orlandi M, Matucci-Cerinic M (2013) Gynaecological symptoms and sexual disability in women with primary Sjögren’s syndrome and sicca syndrome. Clin Exp Rheumatol 31:683–690. https://pubmed.ncbi.nlm.nih.gov/23710558/23710558

[30] Lendrem D, Mitchell S, Mcmeekin P, Bowman S, Price E, Pease CT (2014) Health-related utility values of patients with primary Sjӧgren’s syndrome and its predictors. Ann Rheum Dis 73:1362–1368. https://ard.bmj.com/content/73/7/1362.short 10.1136/annrheumdis-2012-202863 23761688

